# Adolescents, Adults and Rewards: Comparing Motivational Neurocircuitry Recruitment Using fMRI

**DOI:** 10.1371/journal.pone.0011440

**Published:** 2010-07-06

**Authors:** James M. Bjork, Ashley R. Smith, Gang Chen, Daniel W. Hommer

**Affiliations:** 1 Division of Clinical Neuroscience and Behavioral Research, National Institute on Drug Abuse, National Institutes of Health, Bethesda, Maryland, United States of America; 2 Laboratory of Clinical and Translational Studies, National Institute on Alcohol Abuse and Alcoholism, National Institutes of Health, Bethesda, Maryland, United States of America; 3 Scientific and Statistical Computing Core, National Institute of Mental Health, National Institutes of Health, Bethesda, Maryland, United States of America; Kyushu University, Japan

## Abstract

**Background:**

Adolescent risk-taking, including behaviors resulting in injury or death, has been attributed in part to maturational differences in mesolimbic incentive-motivational neurocircuitry, including ostensible oversensitivity of the nucleus accumbens (NAcc) to rewards.

**Methodology/Principal Findings:**

To test whether adolescents showed increased NAcc activation by cues for rewards, or by delivery of rewards, we scanned 24 adolescents (age 12–17) and 24 adults age (22–42) with functional magnetic resonance imaging while they performed a monetary incentive delay (MID) task. The MID task was configured to temporally disentangle potential reward or potential loss anticipation-related brain signal from reward or loss notification-related signal. Subjects saw cues signaling opportunities to win or avoid losing $0, $.50, or $5 for responding quickly to a subsequent target. Subjects then viewed feedback of their trial success after a variable interval from cue presentation of between 6 to17 s. Adolescents showed reduced NAcc recruitment by reward-predictive cues compared to adult controls in a linear contrast with non-incentive cues, and in a volume-of-interest analysis of signal change in the NAcc. In contrast, adolescents showed little difference in striatal and frontocortical responsiveness to reward deliveries compared to adults.

**Conclusions/Significance:**

In light of divergent developmental difference findings between neuroimaging incentive paradigms (as well as at different stages within the same task), these data suggest that maturational differences in incentive-motivational neurocircuitry: 1) may be sensitive to nuances of incentive tasks or stimuli, such as behavioral or learning contingencies, and 2) may be specific to the component of the instrumental behavior (such as anticipation versus notification).

## Introduction

American adolescents suffer substantial morbidity and mortality due to behavioral causes (primarily acts of violence or motor vehicle accidents), even compared to similarly healthy young adults (U.S. Centers for Disease Control). Advances in developmental neuroscience have raised an important question: Might increased adolescent risk-taking be attributable in part to maturational differences from adults (or younger children) in regional brain structure or function? This possibility has profound policy implications [1], and has been invoked not only as justification for graduated drivers licensing, but has also been cited in amicus briefs to the U.S. Supreme Court concerning whether to incarcerate for decades (Pittman v. South Carolina), or even execute (Roper v. Simmons) persons for crimes committed while an adolescent.

Developmental neuroimaging findings have detected structural changes in striatum [2] and frontal cortex [3] across adolescence, where frontocortical gray matter morphology maturation continues into the mid 20 s [4], relatively later than other cortex (reviewed in [5]). This has led to speculation that adolescent risk-taking results in part from immature frontocortical cognitive control neurocircuitry that fails to sufficiently monitor or inhibit risky behavior (e.g. [6], [7]). In particular, an opponent-process theory of adolescent impulsivity posits that subcortical incentive-motivational neurocircuitry in the ventral striatum (VS), including the nucleus accumbens (NAcc) functionally matures sooner than top-down frontocortical behavior control circuitry [8], [9]. This ostensibly results in a problematic imbalance of “go” versus “stop” neurocircuitry during adolescence (relative to younger childhood and adulthood). For example, behavioral tasks have shown a biphasic pattern of risk-taking from young childhood to adulthood- with a peak in risky choice under “hot” (emotion-elicited) experimental conditions during adolescence [10].

Framed in the context of instrumental behavior, adolescent impulsivity could result in part from exaggerated mesolimbic responsiveness to either reward-predictive cues (motivation or orienting), or to reward deliveries (consummation or reinforcement). For example, enhanced responsiveness of VS motivational neurocircuitry toward reward-predictive cues may bias behavior choice toward potentially-rewarding activities, irrespective of potential for a harmful outcome. Enhanced VS responsiveness to reward delivery may promote a greater degree of consumption of risk-laden rewards- such as number alcoholic drinks at a party, or the speed of a car (and resultant “rush”) in a street race. Enhanced VS responsiveness to reward deliveries may also bias future choice toward highly-rewarding but riskier alternatives.

**Figure 1:**
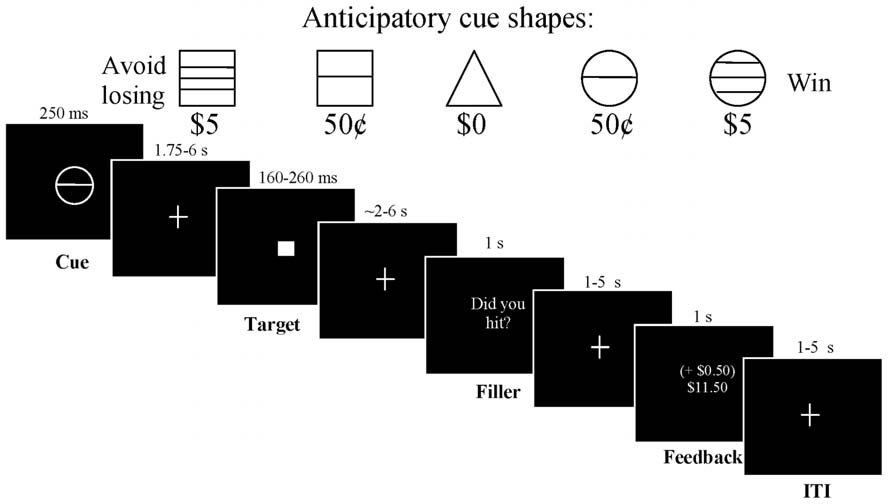
Modified monetary incentive delay (MID) task. Each trial began with presentation of one of five anticipatory cues. The cue signaled the opportunity to either win money (circle series), avoid losing money (square series), or win/lose no money (triangle) by recording a button press while the following white square target was presented on the screen. After target presentation, the subject then waited across a variable delay for notification (feedback) of whether he or she hit the target. During this delay, a lexical filler stimulus (“Did you hit?”) was presented. Intervals between trial stimuli were pseudorandomly varied as indicated, and trials were also separated by a 1–5 s variable intertrial interval (ITI) following each notification.

Characterizing developmental differences in VS functioning is of particular interest because the VS responds to learned reward-predictive cues in primates [11], and in humans can reflect individual differences in motivation by instrumentally-conditioned stimuli [12]. The most widely-adopted probes of human incentive-motivational neurocircuitry are variants of the monetary incentive delay (MID) task (Figure 1), wherein learned cues that signal an imminent opportunity to respond for monetary rewards reliably recruit the VS in proportion to potential reward magnitude [13], [14], [15], [16], [17], [18], [19]. Conversely, the VS is not as robustly recruited by cues for reward deliveries that require no behavioral response in either the MID task [20] or other incentive tasks [21]. In addition, the VS [13], [16], [22] as well as ventral mesiofrontal cortex (mFC) [13], [19], [23], [24], [25] are activated by notification of reward, typically as a contrast with notification of nonreward.

Few neuroimaging studies to date have explored maturational differences in subcortical incentive neurocircuitry between adolescents and adults, and extant findings are mixed. In a preliminary study of developmental differences in VS recruitment by instrumental reward-predictive cues of the MID task [13], adolescents showed reduced right VS recruitment by reward cues compared to adults, with no age group differences in VS or mFC recruitment by reward notifications. In contrast, adolescents showed greater left VS activation by notification of money won in a probabilistic gambling task compared to adults [22]. Similarly, rewarded trials in decision-making tasks elicited a nonlinear developmental pattern of VS recruitment [8], [26]. In the Galvan study [8], once associations between cues and rewarding outcomes had become learned, adolescents showed greater VS activation by rewarding trials compared to responses of adults or younger children. In the Van Leijenhorst study [26], mid-adolescents showed greater VS activation by risky gains than younger children or young adults. Finally, in a slot machine task, where outcomes were predetermined, mid-adolescents also showed more VS activation by reward-predictive cues than younger children and young adults [27].

We note that these age differences in activation reported in most studies were primarily in conjunction with behavior execution or outcome. Conversely, in [8], early VS responses to the initial reward-predictive cue appeared increased in adults relative to adolescents. This underscores the importance of disentangling different components of instrumental behavior. Indeed, using an incentivized anti-saccade task, Geier et al [28] reported that developmental differences in VS recruitment by instrumental behavior varied in directionality *within-task* depending on what component of the instrumental behavior sequence is being assessed. In particular, adults showed relatively greater VS signal ostensibly linked to incentive cue presentation (anticipation), but adolescents later showed greater activation ostensibly elicited by oculomotor response preparation.

If reward notifications were temporally-separated from anticipatory cues, might the MID task reveal greater reward notification-elicited VS recruitment in adolescents compared to adults? Critically, no developmental-comparison fMRI studies to date have featured variable timing between the reward-predictive cue at the start of the instrumental trial and the subsequent reward notification event of that trial, so as to isolate time series signal change [29], [30] elicited by these different components of the instrumental trial. Modified MID tasks with jittered events within the trial, however, have recently shown success in characterizing activation by reward anticipation cues versus notification-elicited feedback [15], [25], [31].

This experiment was intended to advance understanding of maturational differences in incentive neurocircuitry by separately assessing mesolimbic responses to instrumental cues versus mesolimbic responses to instrumental behavior outcomes. It is a modification of our initial study [13], with improved methodology in several aspects. Most importantly, we altered the MID task to include an extended, variable interval between presentation of the response-anticipatory cue and the trial outcome notification. Second, we augmented the statistical power of the variable event timing by sampling the striatum once per second instead of every two seconds. Third, we upgraded from a quadrature head coil to an 8-channel head coil for better signal detection. Fourth, we doubled the sample size. Finally, we adopted a recently-developed, mixed-effects meta/multi-level analysis (MEMA) software designed for outlier-resistant calculation and comparison of group-wise data. Based on our preliminary study [13], we hypothesized that adolescents would show reduced right NAcc activation by reward-anticipatory cues in the MID task, but adolescents would show greater NAcc activation by reward deliveries.

## Results

### Behavioral and affective responses to the MID task

**Figure 2:**
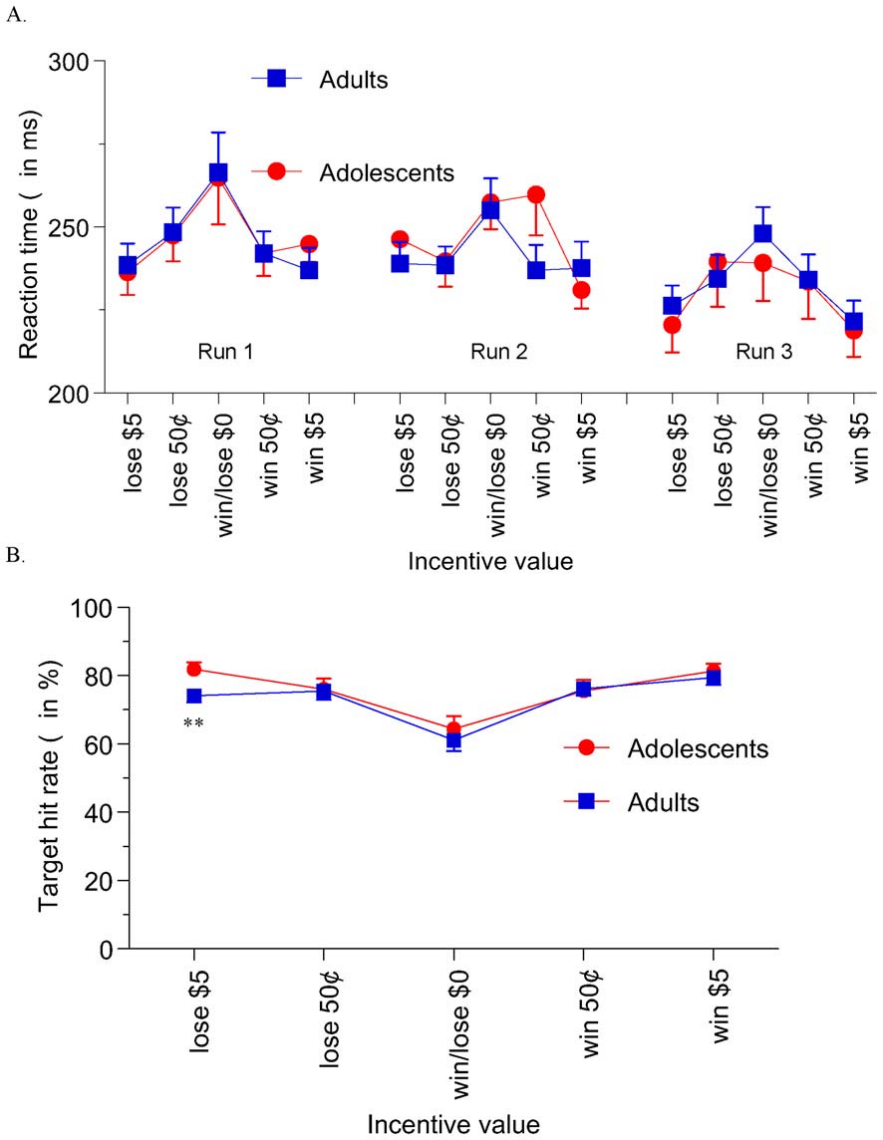
MID task behavior. Mean reaction time (RT) to targets (A) showed significant main effects of trial incentive and time. Specifically, subjects responded more quickly over time, from runs 1 to 3 of the task, and subjects responded more quickly to incentivized, compared to non-incentivized targets. Accordingly, there was a significant main effect of incentive amount on overall task hit rates (B), with a greater proportion of incentivized versus non-incentivized targets hit. There were no main or interactive effects of age group on either RT or hit rates. ** denotes *P*<.05 per simple-effect t-test.

There were no significant main or interaction effects of age group or sex on head-motion correction measures (generated by the volume registration step). No subject moved his or her head more than 3 mm across the whole session or more than 1 mm between successive acquisitions. There was a main effect of time on reaction time (RT) to task targets- in both reward trials (*F*(2,92)  = 7.791, *P*<.001) and in loss-avoidance trials (*F*(2,92)  = 5.263, *P*<.01), where subjects showed faster target RT as the task progressed from run 1 to run 3 (Figure 2, part A). Accordingly, for many subjects we reduced the range of uniform distribution of target display durations between task runs, to promote a 67% hit rate for the entire task. Finally, there was a main effect of incentive magnitude in both reward trials (*F*(2,92)  = 22.996, *P*<.000001) and in loss-avoidance trials (*F*(2,92)  = 21.162, *P*<.000001), where mean RT decreased as incentive magnitudes increased from $0 to 50¢ to $5. There were no main or interactive effects of age group on RT (all *P*≥.3) nor were there any other higher-order interaction effects on RT. Because RT quickened (within-subject) as incentive amounts increased, target hit rates also increased with incentive magnitude, as seen in main effects of magnitude in both reward (*F*(2,92)  = 37.945, *P*<.000001) and loss-avoidance (*F*(2,92)  = 25.136, *P*<.000001) trials (Figure 2, part B). However, there were no main or interactive effects of age group on hit rates.

**Figure 3:**
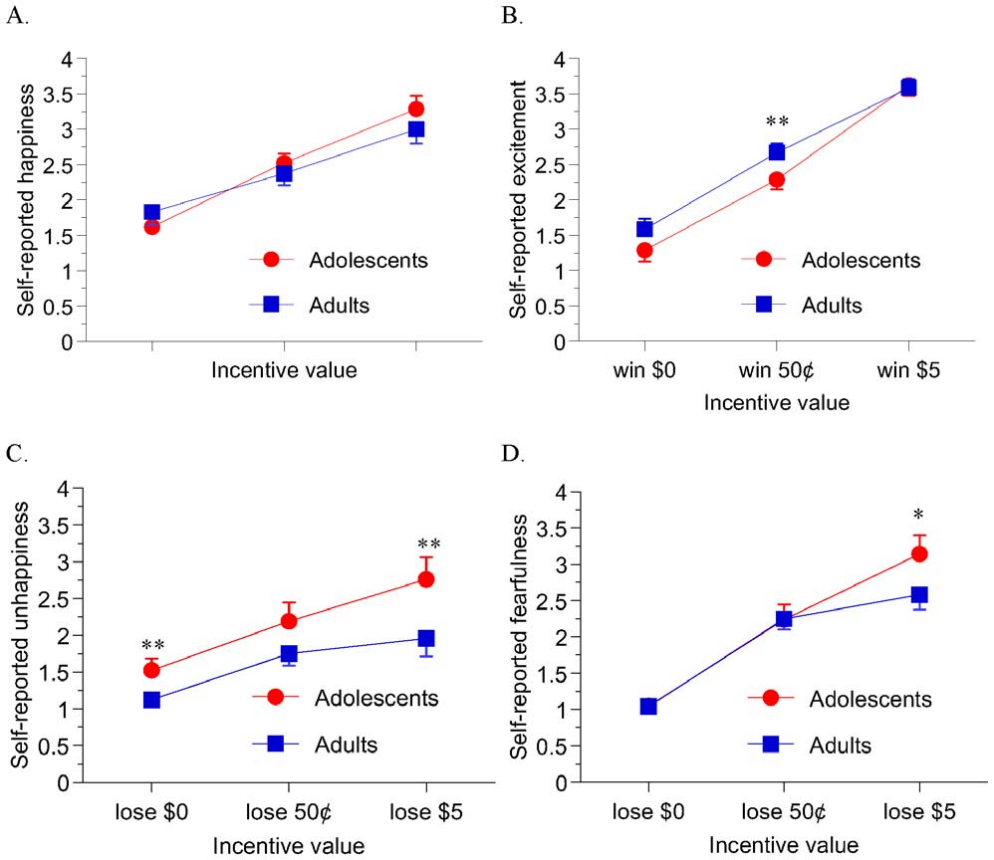
MID task affective ratings. On a post-scan questionnaire, participants reported greater happiness (A) and excitement (B) when seeing anticipatory cues as the potential reward amounts increased. There were no significant main or interactive effects of age group on positive affect ratings. Similarly, subjects reported greater unhappiness (C) and fearfulness (D) as potential loss amounts increased. There were main effects of group (*F*(1,46)  = 5.338, p<.05) on unhappiness ratings across the combined non-incentive and loss-trial types, with greater self-reported unhappiness in adolescents compared to adults. There were no other significant main or interaction effects of age group negative affect ratings. * denotes *P*<.10 and ** denotes *P*<.05 per simple-effect t-test.

There were also significant main effects of incentive magnitude on each of the four affective ratings (Figure 3), where participants reported greater happiness (*F*(2,92)  = 49.033, *P*<.000001) and excitement (*F*(2,92)  = 119.173, *P*<.000001) as potential reward amounts signaled by the cue increased from $0 to 50¢ to $5. There were no significant main or interactive effects of age group on positive affect ratings. Similarly, subjects reported greater unhappiness (*F*(2,92)  = 17.831, *P*<.000001) and fearfulness (*F*(2,92)  = 80.104, *P*<.000001) as potential loss amounts increased from $0 to 50¢ to $5. A main effect of group (*F*(1,46)  = 5.338, *P*<.05) on unhappiness ratings indicated greater self-reported unhappiness (across the combined non-incentive and loss-trial types) in adolescents compared to adults. There were no other significant main or interaction effects of age group on negative affect ratings.

### Statistical maps

#### Reward versus nonincentive anticipation

**Figure 4:**
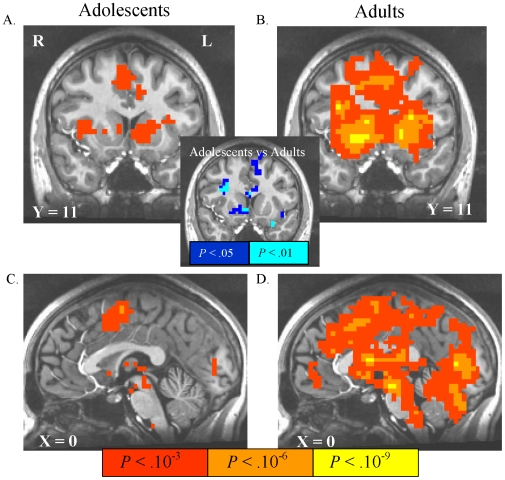
Activation by anticipation of responding for rewards. In these and subsequent statistical maps: 1) all images are right-left reversed per radiological convention, 2) the underlay is a T1-weighted structural image from a representative subject, 3) the Talairach coordinate of the image plane is indicated, 4) illuminated voxels in group-wise maps feature contrast activation that survives false discovery rate (FDR) correction to *P*<.05, and 5) illuminated voxels in the inset group-difference t-statistic maps do not survive FDR correction, but illustrate differences in NAcc recruitment as the structure of *a priori* interest. Anticipation of responding for rewards contrasted with anticipation of responding for no incentive activated portions of ventral striatum (VS) insula, and posterior mesofrontal cortex in both adolescents (A,C) and in adults (B,D). In the inset uncorrected map of the direct voxel-wise age-group difference in activation by this contrast, relatively lower VS activation in adolescents is depicted in cool colors.

Anticipation of responding for potential reward versus anticipation of responding for no incentive activated the VS, bilateral insula, thalamus, mesial occipital cortex, supplementary motor cortex, and voxel clusters that flanked the central sulcus bilaterally in both adolescents and adults (Table 1; Figure 4). Adults, but not adolescents showed suprathreshold activation of the anterior cingulate cortex (ACC) and mesial cerebellum. In the NAcc, which was our *a priori* region-of-interest, the direct voxelwise t-test group difference in activation by this contrast was significant, with reduced adolescent activation relative to adults in right NAcc (Figure 4, inset). There were no brain regions, however, that showed a significant age-group difference that survived FDR correction in the remaining voxels of scan coverage. Results of an exploratory *post hoc* analysis directly contrasting anticipation of potential rewards with anticipation of potential punishments are presented in supplemental Figure S1.

**Table 1:** Activations by anticipatory cues signaling potential rewards versus no incentive.

	Talairach Coordinates			t-value	Uncorrected *P* *
*Adolescents*					
R Nucleus accumbens	5	9	−1	4.646	<.0001
L Caudate head	−9	15	3	4.872	<.0001
L Ventral putamen	−14	9	−5	4.863	<.0001
R Insula	31	15	11	5.262	<.00001
L Insula	−30	14	12	4.270	<.001
L Thalamus	−7	−22	5	5.068	<.00001
R Precentral gyrus	29	−6	50	7.141	<10^−8^
L Precentral gyrus	−34	−22	55	6.698	<.0000001
Supplemental motor area	−1	−4	53	6.085	<.000001
R Superior parietal lobule	27	−64	43	4.694	<.0001
R Cuneus	17	−64	10	4.485	<.0001
L Cuneus	−17	−64	10	4.631	<.0001
Mesial lingual gyrus	−2	−82	1	4.650	<.0001
*Adults*					
R Ventral putamen	16	1	−1	10.86	<10^−11^
L Caudate head	−14	11	0	7.719	<10^−10^
R Thalamus	7	−12	8	7.818	<10^−10^
L Thalamus	−17	−22	1	8.718	<10^−10^
Anterior midbrain	3	−15	−10	9.419	<10^−10^
R Postcentral gyrus	25	−32	59	7.190	<10^−8^
L Precentral gyrus	−37	−22	53	9.252	<10^−11^
Anterior cingulate cortex	−2	15	42	7.102	<10^−8^
Cuneus	2	−72	8	7.962	<10^−9^
Mesial Cerebellum	3	−69	−14	8.074	<10^−9^

*All activations are listed as the local maxima of a cluster, and survive false discovery rate correction to P≤.05 across all voxels encompassed by the scan coverage.

#### Loss avoidance versus nonincentive anticipation

**Figure 5:**
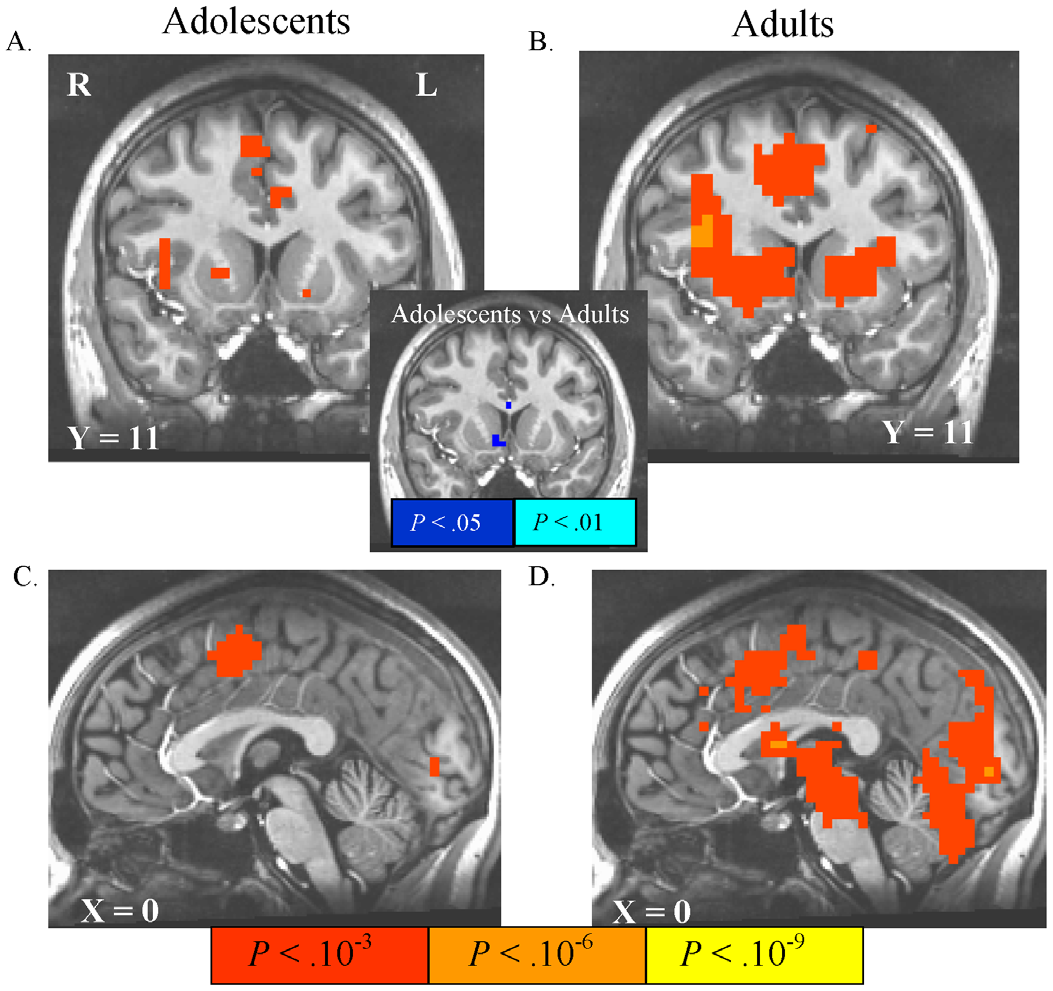
Activation by anticipation of responding to avoid losses. Anticipation of responding to avoid losses contrasted with anticipation of responding for no incentive activated striatal voxels in both adolescents (A,C) and in adults (B,D). In the inset uncorrected map of the direct voxel-wise age-group difference in activation by this contrast, relatively lower VS activation in adolescents is depicted in cool colors.

Anticipation of responding to potentially avoid losses versus anticipation of responding for no incentive activated the VS, lateral thalamus, mesial occipital cortex, supplementary motor cortex, mesial cerebellum, and bilateral pre/postcentral gyri in both adolescents and adults (Table 2; Figure 5). Adults showed a more anterior extent of suprathreshold activation in cingulate cortex, relative to adolescents. The statistical map of the direct voxelwise group difference in this contrast, however, also indicated a decrement in adolescent activation relative to adults in right NAcc (Figure 5, inset). As with potential reward anticipation, there were no voxels across the remaining scan coverage that showed an FDR-corrected age group difference in activation by this contrast.

**Table 2:** Activations by anticipatory cues signaling potential losses versus no incentive.

	Talairach	Coordinates		t-value	Uncorrected *P* *
*Adolescents*					
R Ventral putamen	16	8	−7	4.192	<.001
L Putamen	−15	10	3	3.617	<.001
L Ventral putamen	−14	9	−5	4.863	<.0001
R Insula	39	7	11	5.640	<.00001
L Thalamus	−7	−23	6	3.795	<.001
R Precentral gyrus	38	−7	50	5.624	<.00001
L Precentral gyrus	−38	23	53	4.730	<.0001
Supplemental motor area	0	−1	47	4.838	<.0001
R Middle occipital gyrus	29	−90	8	5.970	<.00001
L Cuneus	−14	−90	−2	4.701	<.0001
Mesial cerebellum	4	−75	−35	4.940	<.0001
*Adults*					
R Putamen	17	1	3	6.414	<.000001
L Ventral putamen	−14	5	−5	6.039	<.000001
R Thalamus	7	−17	2	5.903	<.000001
L Thalamus	−7	−14	1	6.258	<.0000001
Midbrain	7	−22	−9	6.161	<.000001
R Precentral gyrus	29	−18	46	5.024	<.00001
L Precentral gyrus	−39	−21	62	5.925	<.000001
Anterior cingulate cortex	10	8	40	6.232	<.000001
Anterior cingulate cortex	5	35	35	4.952	<.0001
R Superior parietal lobule	29	−50	38	6.192	<.000001
L Superior parietal lobule	−32	−62	46	5.429	<.000001
Cuneus	−6	−86	0	6.885	<.10^−8^
Mesial cerebellum	2	−67	−14	6.165	<.000001

*All activations are listed as the local maxima of a cluster, and survive false discovery rate correction to P≤.05 across all voxels encompassed by the scan coverage.

#### Activation by trial outcome notifications

**Figure 6:**
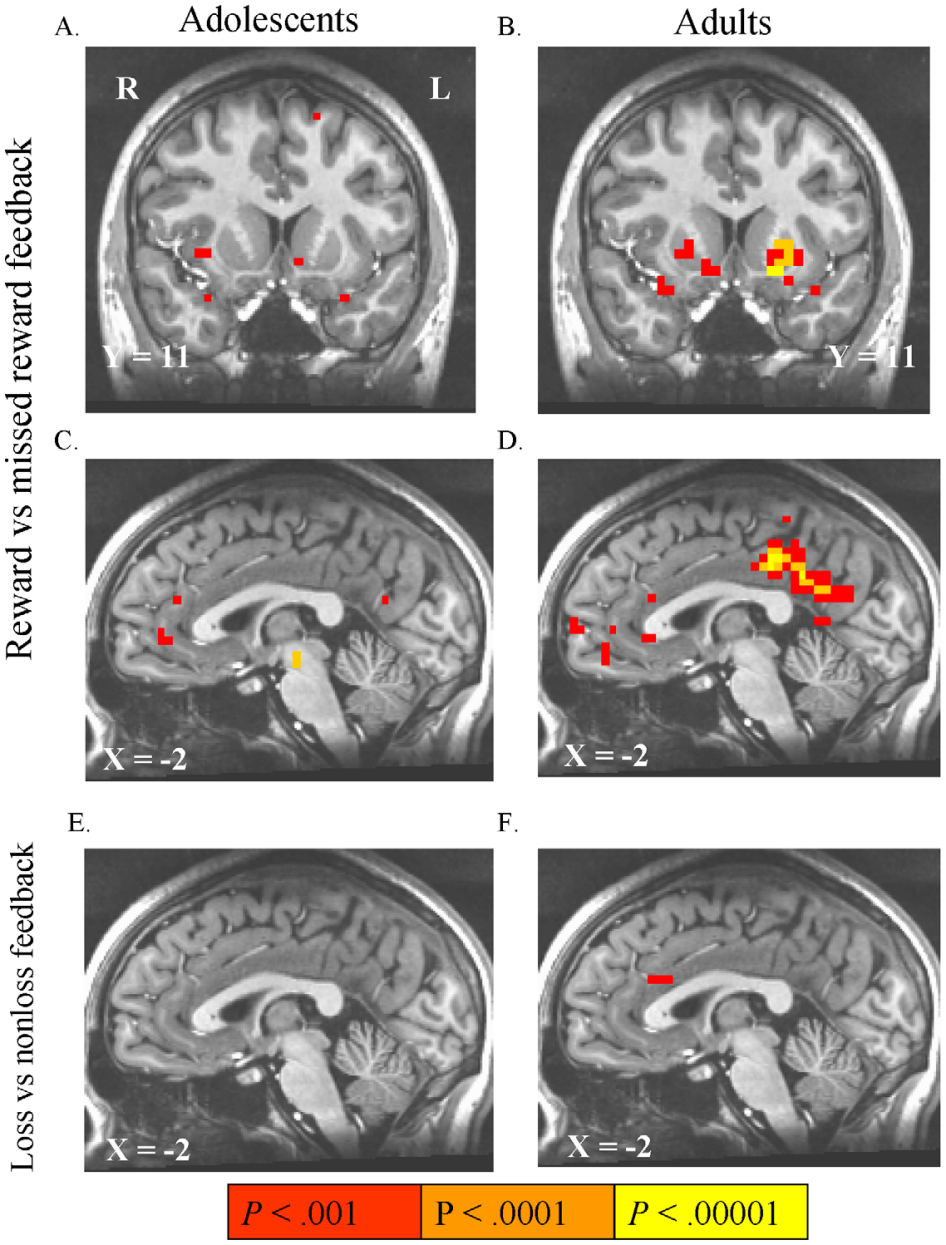
Activation by notification of rewards and losses. Notification of rewards (contrasted with notification of failure to win reward) activated the VS and mesofrontal cortex (mFC) in both adolescents (A,C) and in adults (B,D). Notification of all losses (versus notification of successful loss avoidance) did not activate any voxels above threshold in adolescents (E), but activated anterior cingulate cortex in adults (F).

Notification of rewards (hits) versus notification of nonrewards (misses) in reward trials activated the NAcc, mFC, mesial occipital cortex and amygdala in both adolescents and adults (Table 3; Figure 6). Similar to the anticipation contrasts, no voxels showed an FDR-corrected age group difference in activation by reward notifications. In loss avoidance trials, loss notifications (misses) versus avoided-loss notifications (hits) activated ACC in adults, with no activation in adolescents.

**Table 3:** Activations by reward and loss notifications.

	Talairach Coordinates			t-value	Uncorrected *P* *
***Reward versus nonreward notification***					
*Adolescents*					
R Caudate head	10	14	−3	3.988	<.001
L Nucleus accumbens	−8	9	−5	3.767	<.001
R Amygdala	23	−4	17	5.516	<.00001
L Amygdala	−18	−4	−15	3.618	<.001
R Parahippocampal gyrus	25	−23	−17	7.511	<.000001
L Parahippocampal gyrus	−19	−22	−15	5.027	<.00001
Mesial frontal cortex	5	55	−5	3.531	<.001
Posterior cingulate gyrus	−2	−61	18	4.697	<.0001
R Anterior cingulate gyrus	10	34	20	5.211	<.00001
*Adults*					
R Nucleus accumbens	8	9	−5	4.951	<.00001
L Nucleus accumbens	−14	8	−6	5.511	<.00001
R Amygdala	16	−8	13	5.569	<.00001
R Cuneus	14	−98	5	5.554	<.00001
L Precuneus	−7	−65	19	5.576	<.00001
Mesial frontal cortex	−6	47	−5	5.130	<.00001
Posterior cingulate gyrus	2	−42	37	7.203	<10^−8^
***Loss versus avoided-loss notification***					
*Adolescents*					
No activations					
*Adults*					
Anterior cingulate gyrus	−2	24	24	3.947	<.001

*All activations are listed as the local maxima of a cluster, and survive false discovery rate correction to P≤.05 across all voxels encompassed by the scan coverage.

### Volume of interest (VOI) analyses

#### Cue-elicited anticipatory activation

**Figure 7:**
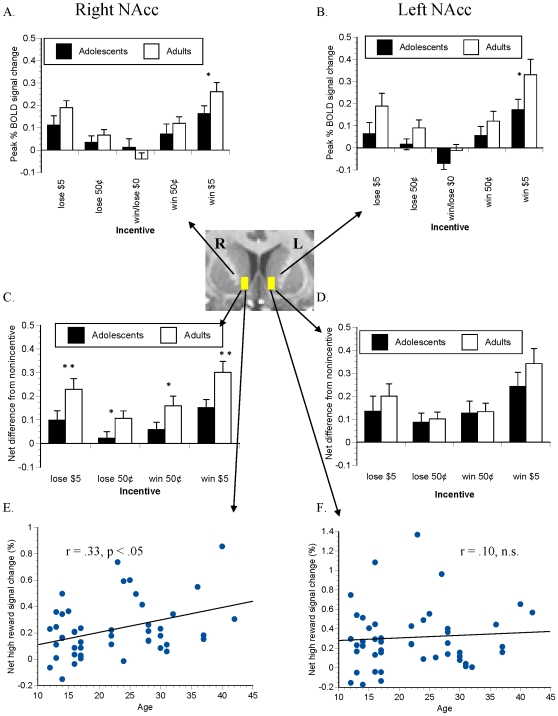
Anticipatory signal change in NAcc VOI. Time series data were extracted from a two-voxel mask in Talairach space in each of right and left NAcc (inset), for each trial type separately. Group mean peak modeled anticipatory signal changes (∼6 s post-cue) are presented as absolute signal change from baseline in parts A and B, and as a net difference from the signal change following presentation of the nonincentive cue (parts C and D). Net signal change elicited by high-reward cues correlated with age in right (E) but not left (F) NAcc. * denotes *P*<.10 and ** denotes *P*<.05 per simple-effect t-test.

Mean peak modeled BOLD signal change in the NAcc VOI masks in all trial types is illustrated in Figure 7, parts A and B. The net signal change (difference from non-incentive trials) in the incentivized trials is illustrated in Figure 7, parts C-F. These main effect of age group did not reach significance (*F*(1,45)  = 2.854, *P*<.10). Simple-effect independent t-tests of net signal change indicated significantly reduced net activation in adolescents. There were main effects of both incentive valence (*F*(1,45)  = 24.786, *P*<.00001) and incentive magnitude (*F*(1,45)  = 34.276, *P*<.000001) on net anticipatory NAcc recruitment, with greater NAcc recruitment by prospective rewards than by prospective losses, and by $5 incentives compared to the 50¢ incentives. In addition, there was also a valence by magnitude interaction effect (*F*(1,45)  = 4.297, *P*<.05), with more magnitude sensitivity in reward trials than in loss-avoidance trials. Complete hemodynamic time-course responses to anticipatory cues are plotted in supplemental Figure S2.

#### Outcome notification-elicited activation

**Figure 8:**
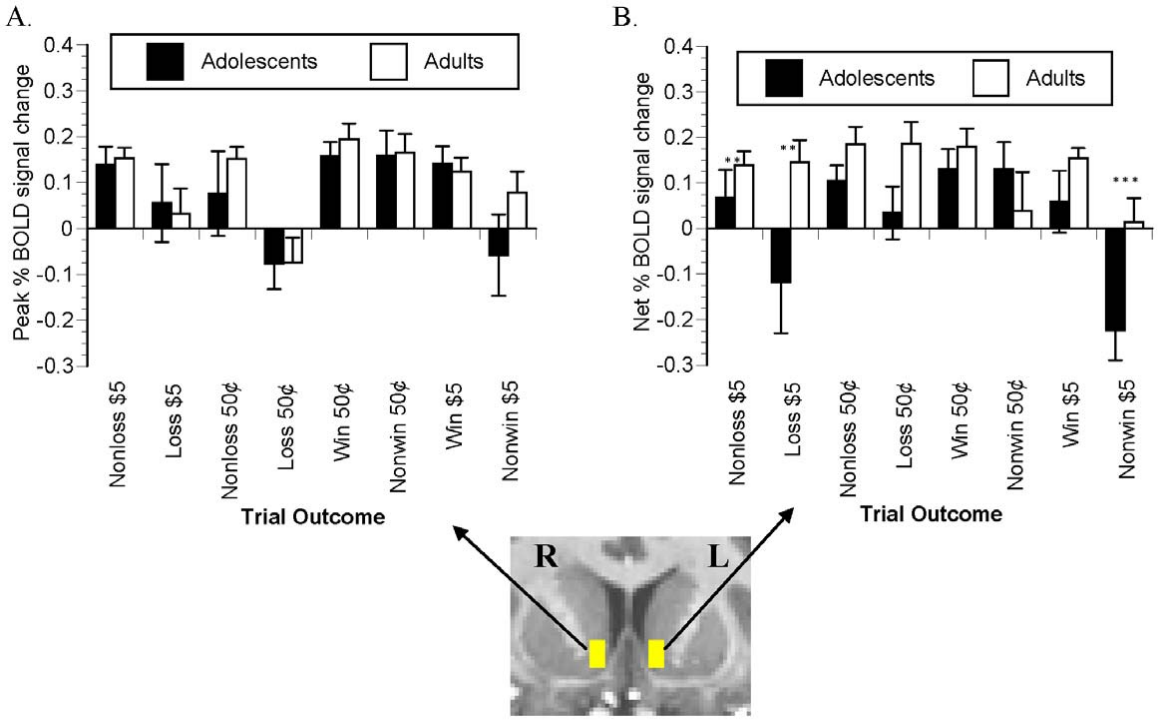
Notification-elicited signal change in NAcc VOI. Trial-outcome-averaged time series data were extracted from each of the right and left NAcc masks (inset). Group mean modeled peak outcome-elicited signal changes (∼6 s post-cue) are presented here as signal change from baseline. * denotes *P*<.10 and ** denotes *P*<.05 per simple-effect t-test.

Peak outcome-elicited signal change in the NAcc VOI masks (Figure 8, parts A and B) indicated a main effect of trial outcome (*F*(1,45)  = 22.823, *P*<.0001), with greater BOLD signal following notification of target hits (rewards or avoided losses) than following misses (missed rewards or losses) overall. A significant magnitude X outcome interaction (*F*(1,45)  = 7.071, *P*<.05) indicated that the outcome-sensitive activation was more pronounced in $5 trials compared to 50¢ trials. Finally, a significant group X magnitude X outcome X side interaction effect (*F*(1,45)  = 4.083, *P*<.05) indicated that in the left NAcc, success-dependence of signal change with increasing incentive amount was more pronounced in the adolescents. Simple effect t-tests of age group differences indicated no difference between age groups in NAcc recruitment by reward notification.

### Developmental correlates of MID task activation

We explored whether potential reward anticipation or reward notification-elicited activation correlated directly with age or with sexual maturation as indexed by Tanner scores. To reduce comparisons, we analyzed activation in high-reward ($5) trials only. Age correlated with net reward-anticipatory signal change (calculated as difference from non-incentive trials) in the right NAcc (Spearman r = .35, p<.05; Figure 7, part E), but not in left NAcc (Figure 7, Part F). When net self-reported excitement about high-reward cues (difference from excitement about nonincentive cues) was entered in a regression model as a second independent variable, NAcc net peak reward-anticipatory signal increase also positively correlated with chronological age across all subjects in right NAcc (Beta = .36, p<.05) but not left NAcc (Beta  = .14, n.s.). In contrast, self-reported excitement over high-reward cues did not partially correlate with reward-anticipatory activation in either left or right NAcc (Beta <.15, n.s.). In an analysis of the adolescents alone, neither chronological age nor Tanner 1 nor Tanner 2 scale scores partially or bivariately correlated with reward-anticipatory or reward delivery-elicited NAcc activation (all Beta/r<.23, n.s.).

## Discussion

We explored maturational differences between adolescents and adults in motivational and consummatory components of incentive neurocircuitry. Both adolescents and adults showed significant recruitment of VS and mFC by the standard contrasts of the MID task, in accord with previous experiments on human incentive processing [8], [12], [14], [15], [16], [17], [19], [20], [23]. We report here some additional evidence that developmental differences in limbic recruitment by instrumental behavior may depend on the component or stage of instrumental behavior [28]. Chiefly, in accord with our hypothesis, we found that adolescents showed mildly reduced activation of the right NAcc by anticipation of responding for gains or to avoid losses, where in the VOI analysis, there was a mild positive correlation across all participants between age and net reward cue-elicited activation in the right NAcc. In contrast with our second hypothesis, adolescents did not show appreciable differences from adults in NAcc or mFC reactivity to reward deliveries. These findings essentially replicate results of our previous developmental comparison using the MID task [13]. Our results were not appreciably affected when 12-year-old subjects (n = 3) were excluded from analysis (supplemental Figures S3 and S4).

An ancillary finding was that suprathreshold activation of ACC by loss outcomes (as a contrast with avoided losses) was present in adults but not adolescents. Despite how the direct group-wise activation difference did not survive FDR correction, we retain mention of this difference as a preliminary finding due to the extensive implication of this portion of ACC in error monitoring [32]. In particular, adolescents have shown decrements relative to adults in ACC recruitment by pre-decision conflict when opting for rewards with a potential for error [6], [7].

Among adolescent participants, neither recruitment of the NAcc by reward cues nor recruitment by reward deliveries correlated with either age or Tanner scores. Indeed, as illustrated in Figure 7 (parts E and F), there was greater variation of NAcc responses to potential reward within age group than between age groups. Extensive individual differences in VS responsiveness to fMRI task rewards has been found in other studies (e.g. [33]). It may be that incentive neurocircuitry is essentially well-developed in the human brain by mid-to late- adolescence (the majority of adolescents were of Tanner stage 4+), with little remaining development-based variance. Surveying children across a wider (i.e. younger) age range or Tanner stage may be necessary to reveal clear developmental trends prior to adulthood.

Collectively, these findings, on the surface, do not generally support the opponent-process developmental account [8], [9] of adolescent risky behavior. We found essentially no evidence for increased mesolimbic responsiveness to either reward-predictive instrumental cues, or to actual reward deliveries in adolescents compared to adults. However, we note that any developmental deficit in behavior control resulting from some combination of overactive reward processing and deficient inhibitory processing would operate in an incentive- or context-specific manner—i.e. when the individual is offered a particular real-world risky incentive. It may be that other incentive paradigms may naturalistically reflect risky incentive scenarios (thus invoking maturationally-deficient dual-processing) better than the MID task.

The MID task features several unique characteristics compared to other incentive paradigms used in children that may explain divergent findings. First, the expected values (contingencies) signaled by anticipatory cues are trained in advance, such there is no discovery or learning in the task, except for discovery of trial-wise success. In particular, adolescents showed increased NAcc responsiveness to rewards of uncertain (secret) magnitude compared to adults [8], whereas subjects in this experiment were explicitly shown the exact (modest) amounts of money they won in a trial. Second, MID task visual stimuli are mundane compared to those of other incentive tasks (e.g. the pirate cartoons of [8] and slot-machine wheels of [27]). We note too that other tasks often feature risky decision-making and waiting for the outcomes of gambles [22], [26], akin to placing a roulette wheel bet, and this is probably more entertaining than a simple MID reaction-time task. Third, the MID task requires unusual vigilance and anticipatory motor preparation- especially for high-incentive targets. Indeed, we cannot rule out that reduced attentional capacity contributed to blunted anticipatory NAcc activation in adolescents. Critically, impaired sleep is common among adolescents [34], and has been linked to deficient striatal recruitment during reward anticipation [35]. In addition, both adults [36] and adolescents [37] with attention-deficit disorder have shown reduced reward-anticipatory activity in the MID task. It can be argued, however, that focused attention is simply one downstream manifestation of motivation, and that adolescents were simply not as motivated as adults to execute the instrumental responses. Finally, we note that in contrast to comparisons between mid-adolescents and very young adults (e.g. [26], [27]), we selected a somewhat older, post-college age group of adults that markedly differs from adolescents in general incidence of behavior-related mortality and morbidity (U.S. Centers for Disease Control).

Taken together with results from our previous experiment [13], these results indicate that if adolescents tend to have greater mesolimbic sensitivity to rewards, this does not generalize to all contexts or tasks. We believe that rather than being a source of confusion, these divergent findings present an intriguing avenue for future research. In particular, if adolescents show reduced motivational neurocircuitry recruitment in the context of mundane work for explicit rewards, but increased activation in the context of more entertaining tasks or non-explicit rewards, this could represent a maturational risk factor for behavior-related mortality and morbidity in adolescence within the domain of reward processing alone—all in the context of reduced top-down executive control. Put differently, in adolescents, there may be unusually great appeal in trying to win $10 racing the adjacent car to the next stoplight as opposed to earning it raking leaves. Of great interest are future experiments that parametrically modulate these different aspects of an incentive task *within-subject*, across the course of a scan, so see if adolescents show greater modulation (interaction) of mesolimbic activation as a function of entertaining task features.

This study has limitations that should be considered. First, this experiment used explicit amounts of money as the incentive. As with any study of groupwise differences in mesolimbic recruitment by monetary incentives, we cannot rule out that observed differences resulted from the amounts of money at stake being more intrinsically valuable in one group compared to another. Therefore, these data may not generalize to other incentives. However, we note that there were no group differences in self-reported excitement or happiness at the prospect of winning money, or in reaction-time to incentivized targets. Also, the directionality of the observed difference in reward-anticipatory activation runs counter to an assumption that the monetary rewards would be *more* valuable to an adolescent compared to an adult wage-earner.

Second, there was a pronounced effect of incentive magnitude on RT, and by extension, on hit rates because the distribution of target durations for each task run was not varied across incentive amounts. It may be that the slower pace of this variant of the MID task made it easier, and enabled maximization of attentional resources of all subjects for the occasional high-value targets. This may also explain the lack of a correlation here between individual differences in self-reported excitement about high-reward cues and NAcc recruitment, which is often found in experiments using the original (briskly-paced) MID task (e.g. [13], [16], [19]) The slow pace of trials, however, was the necessary trade-off in task design to promote separate detection of anticipation- versus notification-elicited BOLD signal. Third, the psychologically healthy adolescents scanned in this experiment are not at particular risk for adverse psychiatric outcomes. Rather, it is youth with histories of conduct disorder or other externalizing symptomatology who are most likely to engage in risky behaviors [38], including substance abuse [39], [40]. Notably, in another recent experiment [19], we found that unmedicated teens with externalizing disorders had significantly greater NAcc activation by notification of rewards and greater NAcc deactivation by missed rewards, compared to age- and gender-matched controls.

Finally, we note that these and other neurodevelopmental brain research findings are merely descriptive and correlational. Accordingly, we can only speculate that observed age-group differences in structure or function of incentive-related brain regions play a role in the increased behavior-related mortality and morbidity of adolescents. It may be that the maturation of incentive neurocircuitry by adolescence is essentially sufficient for rational decision-making, and that psychosocial or cultural factors may underlie increased engagement in risky behaviors among American adolescents [41]. For example, within an economic, expected-utility framework, adolescent risk-taking has been described as rational in the context of social reinforcement contingencies unique to adolescence [42]. However, in light of the extensively-documented maturational differences in structure and function of brain regions extensively implicated in incentive processing and in top-down executive control (reviewed in [43]), we nevertheless raise the possibility that these neurodevelopmental differences may contribute to vulnerability of adolescents to mortality and morbidity to behavioral causes.

In conclusion, this experiment largely replicates findings from our initial investigation [13], where adolescents showed reduced recruitment of the right NAcc by reward-predictive cues, but similar activation of mesolimbic incentive-motivational neurocircuitry to reward notifications. In addition, we found significant recruitment of ACC by notification of losses in adults but not adolescents. Future experiments could expand on these findings by artificially manipulating instrumental trial outcomes (such as omissions of expected rewards), and could reconcile divergent findings of maturational differences in incentive processing by modulating stimulus or other features of incentive tasks.

## Methods

### Ethics Statement

Recruitment and testing procedures were conducted in accord with the Declaration of Helsinki, and were approved by the Institutional Review Board of the National Institute on Alcohol Abuse and Alcoholism (NIAAA). All subjects, including parent informants, provided written informed consent to participate.

### Subjects

Adolescent (n = 24; age 12–17, mean 14.8±1.8; 12 males) and adult (n = 24; age 22–42, mean 29.3±5.7; 12 males) controls were recruited using public internet and print media advertisements. Adolescents participated along with a parent, whose role was to provide medical and psychiatric history information about the adolescent during screening. Subjects were right-handed, with no significant medical illness as determined by physical examination, medical history interview, and clinical chemistry profile. Psychotropic drug abstinence was assessed with a urine drug screen. Adult applicants for the study were administered a structured clinical interview for DSM-IV, and adolescent applicants were jointly assessed with both self-report and parent interviews using the structured Diagnostic Interview for Children and Adolescents (DICA)[44] for DSM-IV. Any Axis I diagnosis was exclusionary. Finally, adolescents self-completed the Tanner scale of physical maturation in a private room equipped with a large mirror. The mean Tanner scores were 3.75±1.07 (SD) for item 1 (breast and testicular maturation) and 4.125±0.95 (SD) for item 2 (pubic hair growth). Age correlated significantly with Tanner 1 (Pearson r = .73, p<.0001) and Tanner 2 (Pearson r = .70, p = .0002) scale scores.

### Monetary Incentive Delay (MID) task

Stimuli were presented on a screen at the foot of the scanner bed by a projection monitor, and viewed using a head coil mirror. Subjects viewed pseudorandomly-presented trials. Each trial was comprised of four temporally jittered events that were spaced, on average, 4 s apart: anticipatory cue presentation, target presentation, a lexical filler stimulus, and success-dependent feedback (Figure 1). Feedback was then followed by a variable intertrial interval. Subjects were instructed to respond on a button box while each trial's target was displayed. Subjects could win money or avoid losing money for pressing during target presentation.

First, one of five anticipatory cue shapes (which defined the trial type) was presented for 250 msec. Reward cues (circles) signaled that if the subject responded during the subsequent target presentation, he or she would win 50¢ (18 trials) or $5 (18 trials). Similarly, loss-avoidance cues (squares) signaled the possibility of losing either 50¢ or $5 (18 trials) if the subject did not respond to the subsequent target while it was presented. Cues signaling nonincentive outcomes (18 trials; triangles) were also presented, and subjects were instructed to respond to the target, but that trial outcomes would not alter their winnings. Each cue was replaced by a fixation crosshair for a uniformly-distributed variable interval (1750–5750 msec). Second, a white target square was presented for a variable length of time (180–280 msec) and replaced by a crosshair for 1720–5820 msec. Third, a lexical filler stimulus, the question “Did you hit?” was presented for 1 s, followed by a crosshair for 1–5 s. The trial then concluded with feedback (1 s duration), which notified participants of whether they had won or lost money during that trial and also displayed their cumulative earnings. The filler stimulus varied in timing of its onset, and was included to help subjects maintain attention in the task across an extended delay between target response and feedback. Following the trial feedback, there was a variable interval (1–5 s) before the cue of the next trial.

Prior to scanning, subjects were shown an envelope containing the cash they could earn in the task, and were read an instruction script which defined the consequences signaled by the anticipatory cues, and informed the subject that he or she would actually win in cash the sum of task earnings across the three runs of the task. Then, during a 5-minute practice session, reaction times to targets were covertly measured, and a distribution of target presentation durations was set for the scan task such that each participant would likely succeed on ∼66% of trials during the scan. Once in the scanner, each participant engaged in three runs of the MID task (∼7 min each), followed by a structural scan (described below) for anatomical colocalization. Following the scan, subjects rated on four-point scales of how “excited,” “happy,” “fearful,” and “unhappy” they felt when they saw each of the task cues. Subjects were then paid their task earnings. Subjects also received $100 compensation for lost time during the psychiatric and medical screening visit and $80 compensation for the MRI visit.

### FMRI acquisition

Imaging was performed using a 3 T General Electric MRI scanner (General Electric, Milwaukee, WI) and an 8-channel head coil. The 90 trials of the MID task were administered across three runs of functional scans. Each run lasted 490 s, and used a T2*-sensitive echoplanar sequence with a repetition time (TR)  = 1000 msec, echo time (TE)  = 40 msec, flip  = 90°. The initial 12 volumes of each run were discarded, and the MID task began with the initial trial cue at the 13^th^ volume. In each volume, we collected sixteen 5.0-mm-thick contiguous saggital slices centering on the intrahemispheric fissure. This montage sampled the anteroposteral extent of striatum once per second- twice the rate of most incentive neuroimaging experiments, including [13]. This rapid sampling rate was intended to maximize statistical power in regression of the time series for anticipation- versus feedback-elicited activation. Coverage included all mesofrontal gray matter extending bilaterally from the intrahemispheric fissure, the entire width of the putamen, and all midbrain structures. In-plane resolution was 3.75×3.75 mm. Structural scans were acquired using a T1-weighted sequence (TR, 100 msec; TE, 7 msec; flip, 90°), which facilitated coregistration of functional data. Each subject's head was restrained with a fabric forehead strap and a series of shaped cushions wedged into the head coil.

### FMRI analysis

#### Preprocessing

Blood Oxygen-Level Dependent (BOLD) signal was analyzed using Analysis of Functional NeuroImages (AFNI) software [45]. Briefly, individual time-series datasets were time-shifted to compensate for non-simultaneous slice acquisition, warped out into Talairach stereotactic space as 3.75 mm isotropic voxels, corrected for head motion, and spatially smoothed to a uniform 8 mm full-width half maximum in brain voxels. Processed time series were modeled with canonical gammavariate hemodynamic responses time-locked to anticipatory cues, targets, and trial outcome notifications. Canonical hemodynamic responses were scaled to 100 so that beta weights (partial correlations) would be equivalent to percent-signal-change. The drifting effect in the signal was fitted with extended polynomials for each run. The jittered stimulus timing of targets enabled modeling out activation related to motor responses. This analysis centered on the four linear contrasts of event-related signal change (hereafter “contrasts”) typically calculated for the MID task: 1) high and low reward vs nonincentive anticipatory cues, 2) high and low loss avoidance vs nonincentive anticipatory cues, 3) reward vs nonreward outcomes in reward trials, and 4) loss versus nonloss outcomes in loss avoidance trials. The regression analysis incorporated correction for the temporal autocorrelation of voxel-wise noise (AFNI program 3dREMLfit).

#### Groupwise and between-group statistical mapping

We have found that VS signal is prone to individual morphology- and scan-based differences in sinus susceptibility artifact, where the NAcc in particular resides at the margin of robust BOLD signal detection. To better accommodate this, instead of a standard ANOVA, groupwise and group-difference maps were calculated in AFNI using recently-developed software, 3dMEMA (http://afni.nimh.nih.gov/sscc/gangc/MEMA.html ), with a linear mixed-effects multilevel model that incorporates both within-subject and cross-subjects variability. Activations in group-wise maps are reported at the maxima of activated voxel clusters, where voxel-wise significance was controlled by the false discovery rate (FDR) set to a false-positive *P*<.05 across the entire scan coverage (search volume). These significant activations are also displayed graphically with a threshold of voxelwise significance set at p<.001. In statistical mapping, direct voxelwise activation differences between age groups are only described in mesolimbic structures previously implicated in this or similar incentive fMRI tasks. In voxels outside the NAcc (our *a priori* structure of interest), group differences are considered statistically significant only if the t-statistic of the group difference survives FDR correction.

#### Volume-of-interest (VOI) analysis of NAcc signal change

We further characterized task-elicited signal change in VOI analyses of the NAcc, which is consistently recruited by the MID task [13], [16], [17], [37]. Each subject's hemodynamic responses were: 1) trial-averaged, 2) modeled and corrected for low-frequency baseline drifts as per the core regression analysis, and 3) passed through a mask in each of the left and right NAcc, and in mFC. To avoid circularity of statistical inference [46], [47], the masks were not localized based on observed contrast activation, but rather were anatomically localized *a priori*. This was a two-voxel mask comprised of the 3.75 mm cubic voxel that corresponded to activation maxima or VOI placement in previous reports (Talairach±8, 11, 0), along with the adjacent voxel located ventrally at the junction of caudate and putamen [48](Figure 7, inset). Visual inspection of this mask overlaid atop Talairach-warped structural images indicated that these voxels were localized almost entirely or entirely in ventromesial striatal gray matter in all but one subject (an adolescent, who was thus excluded from VOI analysis of NAcc signal change).

For incentive-anticipatory activation, peak modeled signal change (∼6 s lag after cue presentation) was analyzed in a mixed-model analyses of variance (ANOVA) across the left and right NAcc masks. Reward-anticipatory BOLD responses were analyzed as the net peak signal difference from the non-incentive control. Incentive magnitude (50¢, $5) incentive valence (rewards, losses) and side (left, right) were within-subject factors, and group (adolescents, adults) the between-subject factor. Finally, we analyzed modeled outcome notification-elicited peak signal change (in incentivized trials) in a mixed-model ANOVA. In these analyses, outcome (hit, miss) added as an additional within-subject variable. This analysis focused on main or interaction effects of trial outcome. Due to the small number of miss events in each trial type singly, investigation of higher-order interactions with outcome was restricted to interaction effects where misses were consolidated across gain and loss valences, such that we did not consider higher-order interactions with both valence and magnitude.

### Behavior Analysis

We performed mixed-model analyses of variance of affective ratings, hit rates, and reaction times (RT) in each of reward trial series and loss-avoidance trial series. The non-incentive trial data (as the control condition) was incorporated twice, once in each of the reward and loss-avoidance trial analyses. Therefore, incentive magnitude (0, 50¢, or $5) was the within-subject factor, and group (adolescents and adults) was the between-subject factor. For the analysis of RT, time (task runs 1–3) was added as an additional within-subject factor.

## Supporting Information

Figure S1 (1.75 MB TIF)

Figure S2 (0.88 MB TIF)

Figure S3 (2.69 MB TIF)

Figure S4 (0.78 MB TIF)
