# Supplementary figures and images for: Adolescents, Adults and Rewards: Comparing Motivational Neurocircuitry Recruitment Using fMRI
Source: PLoS One. 2010 Jul 6;5(7):e11440. doi: 10.1371/journal.pone.0011440 (PMC2897849; doi:10.1371/journal.pone.0011440)

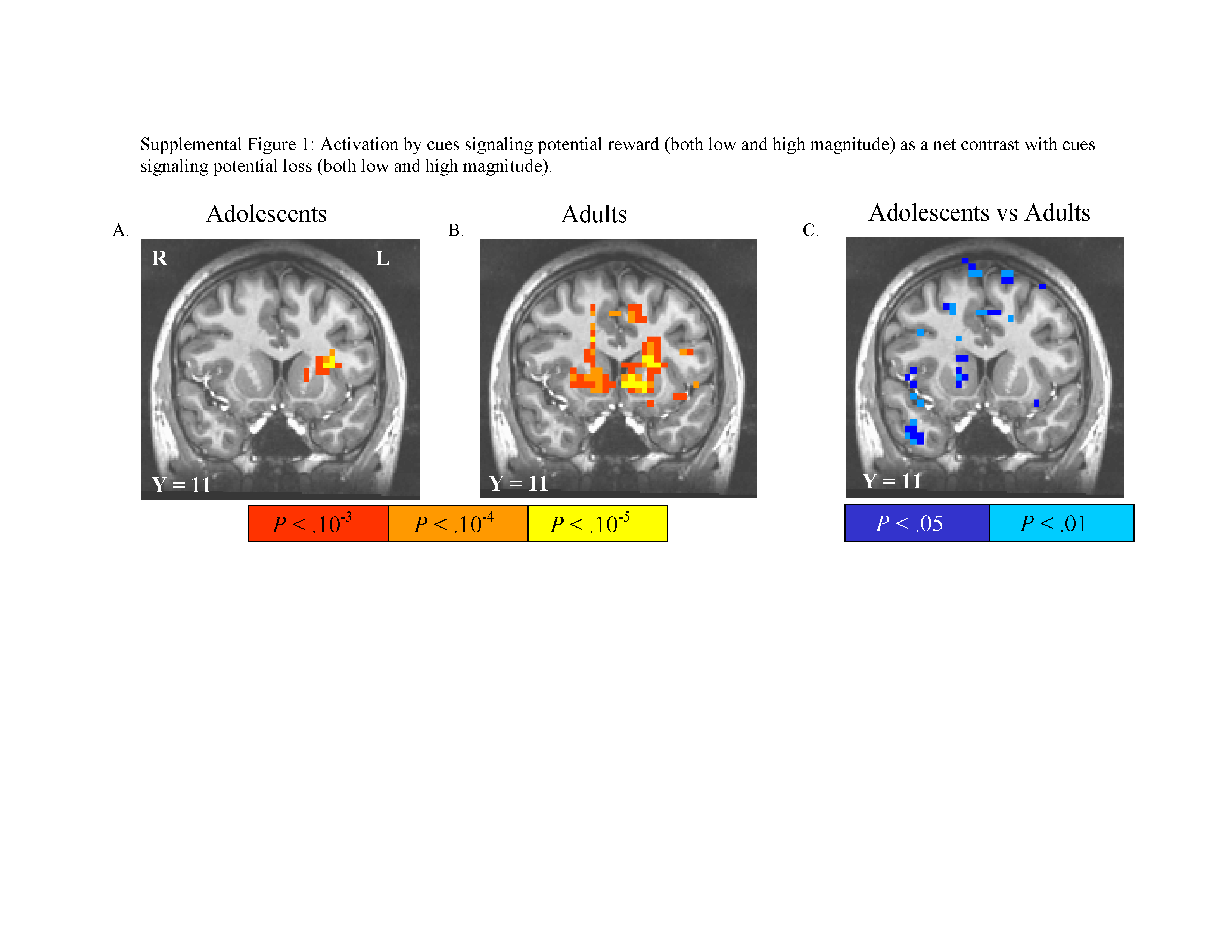

Supplement: Figure S1 — (1.75 MB TIF) [file pone.0011440.s001.tif]

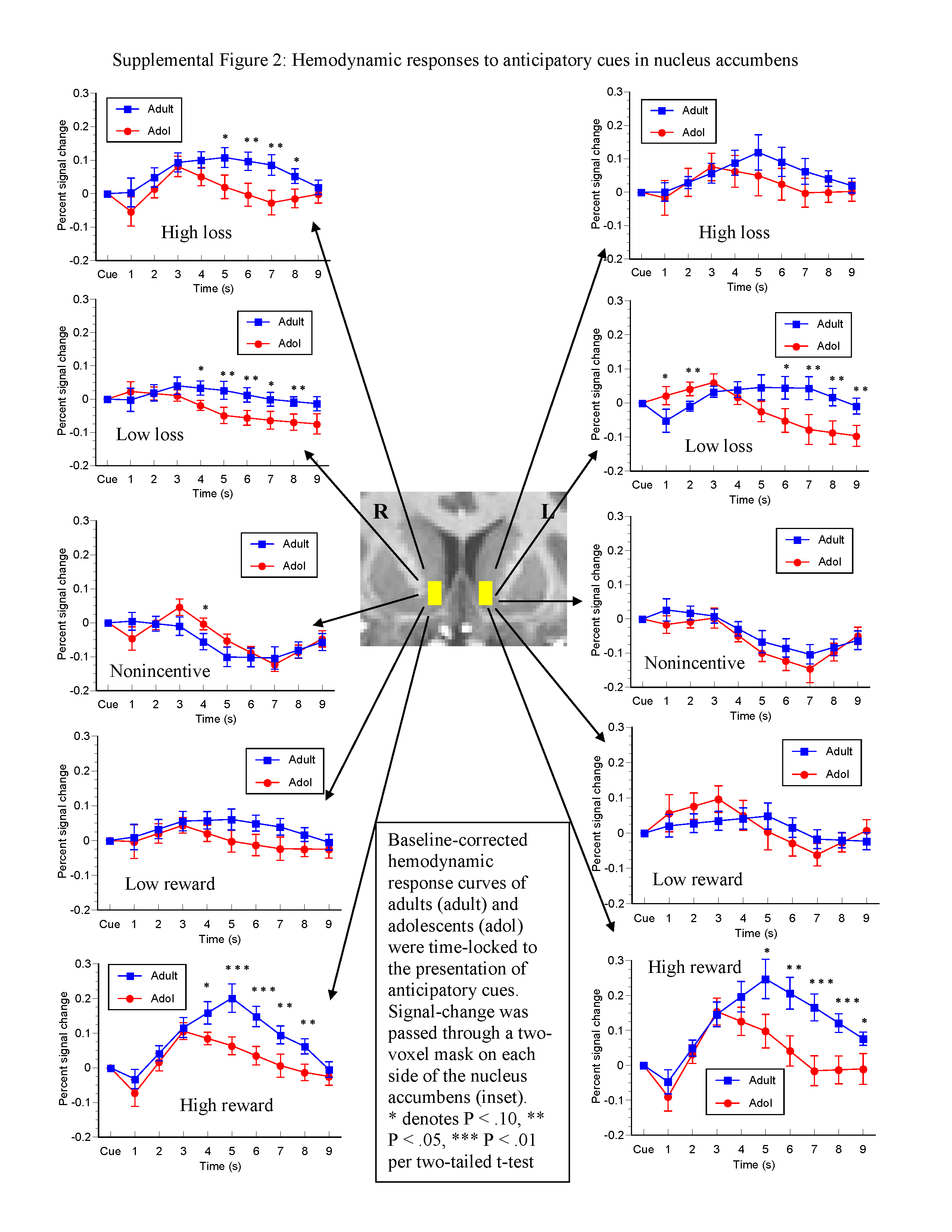

Supplement: Figure S2 — (0.88 MB TIF) [file pone.0011440.s002.tif]

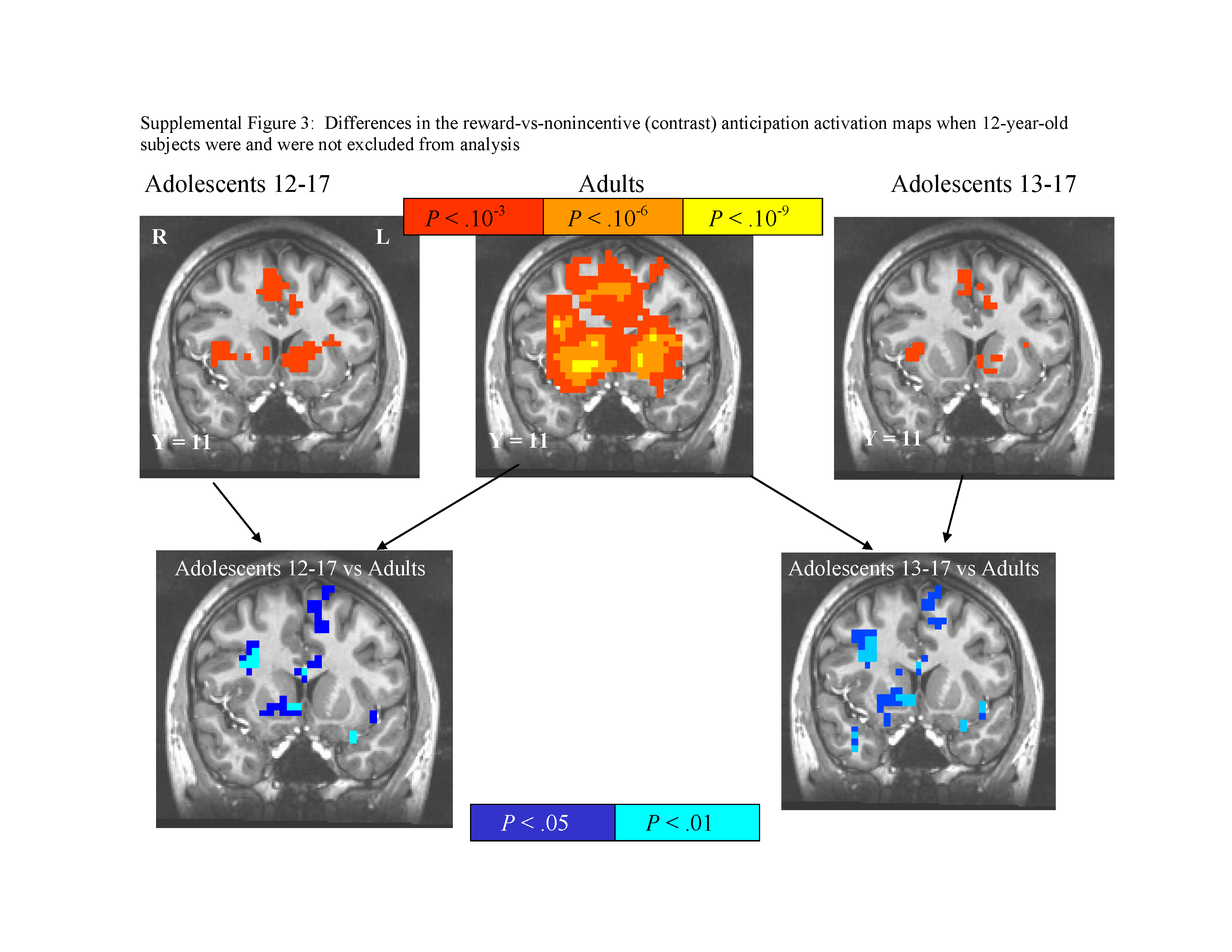

Supplement: Figure S3 — (2.69 MB TIF) [file pone.0011440.s003.tif]

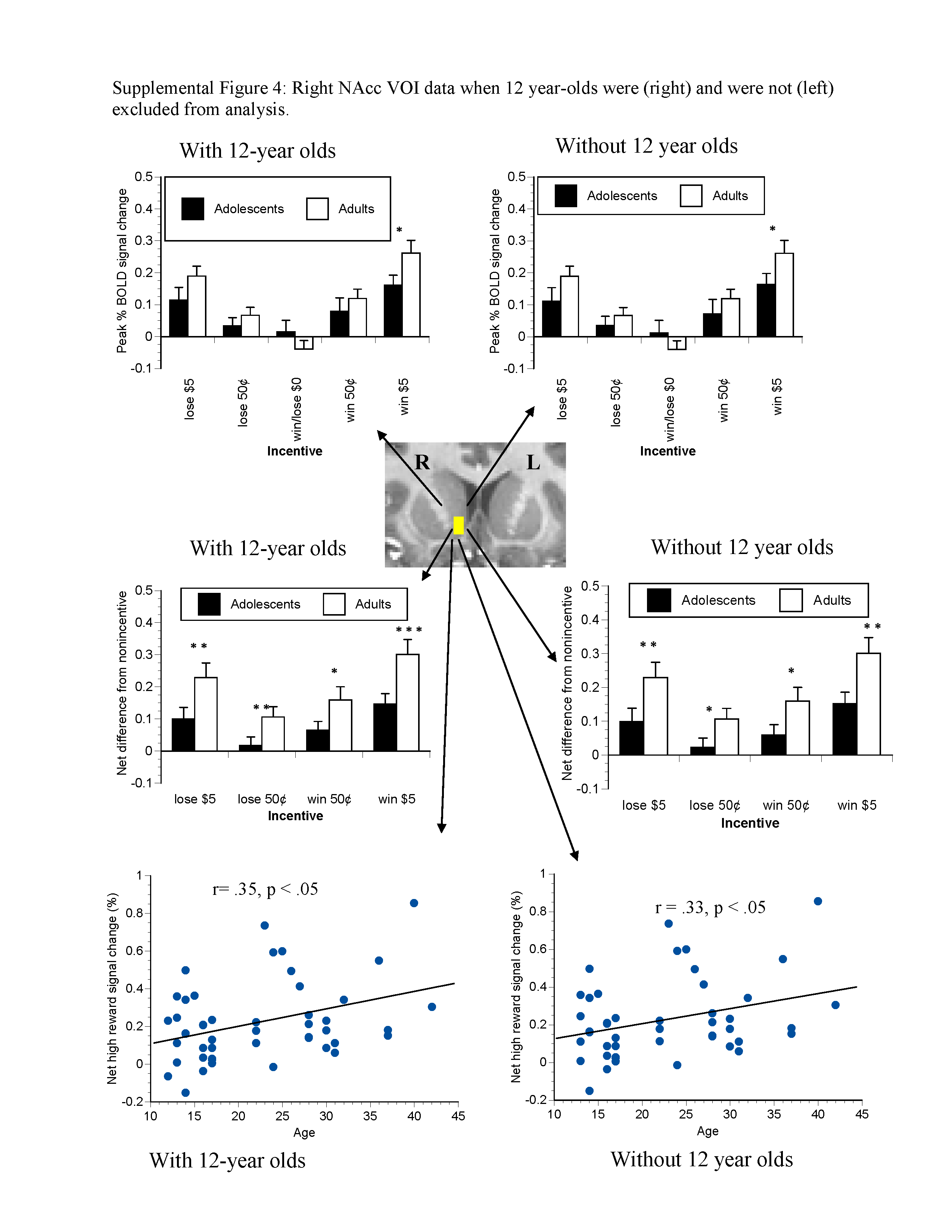

Supplement: Figure S4 — (0.78 MB TIF) [file pone.0011440.s004.tif]
